# Type-Specific Cell Line Models for Type-Specific Ovarian Cancer Research

**DOI:** 10.1371/journal.pone.0072162

**Published:** 2013-09-04

**Authors:** Michael S. Anglesio, Kimberly C. Wiegand, Nataliya Melnyk, Christine Chow, Clara Salamanca, Leah M. Prentice, Janine Senz, Winnie Yang, Monique A. Spillman, Dawn R. Cochrane, Karey Shumansky, Sohrab P. Shah, Steve E. Kalloger, David G. Huntsman

**Affiliations:** 1 Department of Pathology and Laboratory Medicine, University of British Columbia, Vancouver, British Columbia, Canada; 2 Genetic Pathology Evaluation Centre, University of British Columbia and Vancouver General Hospital, Vancouver, British Columbia, Canada; 3 Centre for Translational and Applied Genomics (CTAG), BC Cancer Agency, Vancouver, British Columbia, Canada; 4 Department of Obstetrics & Gynecology, University of Colorado, Anschutz Medical Campus, Aurora, Colorado, United States of America; 5 Department of Molecular Oncology, BC Cancer Agency Cancer Research Centre, Vancouver, British Columbia, Canada; Kinghorn Cancer Centre, Garvan Institute of Medical Research, Australia

## Abstract

**Background:**

Ovarian carcinomas consist of at least five distinct diseases: high-grade serous, low-grade serous, clear cell, endometrioid, and mucinous. Biomarker and molecular characterization may represent a more biologically relevant basis for grouping and treating this family of tumors, rather than site of origin. Molecular characteristics have become the new standard for clinical pathology, however development of tailored type-specific therapies is hampered by a failure of basic research to recognize that model systems used to study these diseases must also be stratified. Unrelated model systems do offer value for study of biochemical processes but specific cellular context needs to be applied to assess relevant therapeutic strategies.

**Methods:**

We have focused on the identification of clear cell carcinoma cell line models. A panel of 32 “ovarian cancer” cell lines has been classified into histotypes using a combination of mutation profiles, IHC mutation-surrogates, and a validated immunohistochemical model. All cell lines were identity verified using STR analysis.

**Results:**

Many described ovarian clear cell lines have characteristic mutations (including *ARID1A* and *PIK3CA*) and an overall molecular/immuno-profile typical of primary tumors. Mutations in *TP53* were present in the majority of high-grade serous cell lines. Advanced genomic analysis of bona-fide clear cell carcinoma cell lines also support copy number changes in typical biomarkers such at *MET* and *HNF1B* and a lack of any recurrent expressed re-arrangements.

Conclusions: As with primary ovarian tumors, mutation status of cancer genes like *ARID1A* and *TP53* and a general immuno-profile serve well for establishing histotype of ovarian cancer cell We describe specific biomarkers and molecular features to re-classify generic “ovarian carcinoma” cell lines into type specific categories. Our data supports the use of prototype clear cell lines, such as TOV21G and JHOC-5, and questions the use of SKOV3 and A2780 as models of high-grade serous carcinoma.

## Introduction

Ovarian cancer is a diverse set of diseases and amongst the most clinically significant, epithelial ovarian cancers (EOC), at least five distinct entities exist [1]–[9]. At a broad level, the terms type I and type II EOCs are often applied, wherein high-grade serous carcinomas (HGSCs) are type II and all other histologies are type I cancers [8]. However, even within type I, distinct entities exist, namely low-grade serous carcinoma (LGSC), endometrioid carcinoma (ENOCa), clear cell carcinoma (CCC) and mucinous carcinoma (MUC). There is significant data suggesting that a majority of HGSC originate from fallopian tube epithelium [1], [10]–[13], while low-grade serous tumors are generally still thought to arise from the ovarian surface epithelium – though this relationship is being questioned [7], [14]. ENOCa and CCC tumors occur in a background of endometriosis and could represent a spectrum of displaced, malignant endometrium [15]–. Finally, mucinous tumors are exceedingly rare and their true origin is difficult to ascertain with subgroups of distinct histology. Their resemblance to other mucinous epithelial malignancies, most notably gastric cancers, has added to the confusion of their origin [3],[21]–[23].

Clinical responses and epidemiological differences are also apparent between histotypes. High-grade serous cancers show the best initial response rates to the current standard chemotherapy regime of platinum and taxanes [24], [25]. Familial *BRCA1/BRCA2* mutations also appear largely restricted to this histology [26]–[28]. Conversely, the minor histotypes tend to occur in younger patient populations and more frequently present at lower stage [29]–[31]. A list of some of the more distinguishing features between histotypes types is given in Table 1.

**Table 1:** Discriminating Features Of The Five Major Histotypes Of Ovarian Carcinoma.

	Clear Cell Carcinoma	Endometrioid Carcinoma	Mucinous Carcinomas (& Mucinous Borderline Tumors)	Low-Grade Serous Carcinomas (& Serous Borderline tumors)	High-grade serous carcinoma
**Presentation**	Presents at younger age and low stage (pelvic mass) [4], [29]–[31]	Presents at younger age (than HGSC) [4], [29]–[31]	Presents at younger age (than HGSC) [4], [29]–[31]Histopathological similarity to gastric carcinomas (intestinal type) [1], [8], [31]	Presents at younger age (than HGSC) [4], [29]–[31]	Presents at older age (than other histotypes) and high stage (ascites common) [4], [8], [29]–[31]
**Precursors**	Associated with Endometriosis [1], [8], [16], [82]	Associated with Endometriosis [1], [8], [16], [82]	Potential link to Walthard cell nests [83]	Association between ovarian surface and fallopian tube epithelium is unclear [14]	Significant subset associated with serous tubal intraepithelial carcinoma (STIC) [1], [8], [11], [84]
**Genetics, Genomics & Biomarkers**	*TP53* wild-type [4], [15]	*TP53* mutations rare [4]	*TP53* wild-type (borderline)*TP53* mutant (∼1/2 of carcinomas) [4], [8]	*TP53* wild-type [4], [8]	*TP53* mutant (virtually ubiquitous, >96%) [9], [85]
	Negligible occurrence of (germline) *BRCA1/2* mutations [26]–[28], [86]	Negligible occurrence of (germline) *BRCA1/2* mutations [26]–[28], [86]	Negligible occurrence of (germline) *BRCA1/2* mutations [26]–[28], [86]	Frequency of BRCA1/2 mutations presumed low	Germline and somatic BRCA dysfunction/high proportion of hereditary (germline) *BRCA1/2* mutation carriers [9], [26]–[28], [86]
	High frequency of *ARID1A* and *PIK3CA* mutations; frequent loss of *PTEN* expression; near ubiquitous expression of HNF1B [15], [16], [45]	High frequency of *ARID1A* mutations; Moderate frequency of *PIK3CA*, *CTNNB1*, and *PTEN* (loss/LOH) mutations [16], [45]	High frequency (55–75%, carcinoma-borderline) of *KRAS* mutations *(ras-pathway mutation almost exclusively KRAS); *Frequent (19%) of high-level *ERBB2* amplification [22]	High frequency mutually exclusive RAS-pathway mutations (*KRAS, BRAF, NRAS,* or *ERBB2*) typical of borderline serous tumors [5], [8], [10], [66]	Complex karyotypes suggestive of a period of massive genomic instability [9], [87]
**Treatment Response and Outcomes**	Higher frequency of thromboembolic complications [15], [88] Low stage outcome better than (stage matched) HGSC; poor initial response to therapy and worse high stage outcomes (vs. HGSC) [15], [89]	Typically longer interval to progression or death than HGSC (confounded by stage). Stage matched analysis (Stage III) suggests little difference in outcome to HGSC [90]	Overall favorable (due to prevalence of low-stage disease), however very poor outcome on recurrence [31], [43]	Poor response to current treatment standards (Platinum/taxane) [91], [92]	Good initial response rates to current treatment standards (Platinum/taxane); relapse and eventual treatment failure is common [4], [24]

Regardless of origin or histological similarities and differences, biomarker and genomic studies have been successfully used to distinguish each histotype and may represent a far more biologically relevant basis for classifying and subsequently treating EOCs. Although this concept is well-accepted, and gaining traction on becoming a new clinical standard, ambiguous cell line models perpetuated through molecular biology bench research hamper the development of tailored type-specific therapies. Those using bench experiment model systems must recognize that, like primary cancers, the models used to study these diseases must also be stratified. Although biochemical studies can generate useful information from using a variety of unrelated model systems, disease specific studies need to apply cellular context. The vast majority of research employing functional studies on “ovarian cancer” cell lines does not properly ascertain the background of their model systems. Resulting conclusions may be difficult to interpret and the value of potential therapeutic targets may be questionable as is the true relevance to a particular disease.

Cell line studies of ovarian cancer have been severely hampered due to the lack of proper annotation of “ovarian” carcinoma cell lines. Once in culture, cells no longer have easily identifiable morphological traits to aid in histological classification. Additionally, human error, mislabeling and the generic feature of “epithelial-like” cell lines have also led to mix ups of cell lines and contamination which has resulted in un-interpretable data [32], [33]. In the post-genome era, biomarkers and genomic features for ovarian carcinoma subtypes are very well established. Screening techniques to assay biomarkers and verify genomic features are also widely accessible. Here, we present a panel of biomarkers and molecular features across 32 commonly used and in-house derived ovarian carcinoma cell lines. Our initial goal was to establish a bona-fide list of CCC cell lines for our own research program, however we propose establishing type-specificity for these cell lines should became the new standard in planning and executing experiments around any study on epithelial ovarian carcinoma.

## Methods

### Cell culture

Cells were maintained in a humidified incubator at 37C with 5% CO_2_. See Table S1 for a list of cell lines, culture conditions and contributing labs and repositories. Some cell lines were derived in-house (labeled with “VOA#”) through continuous *in vitro* culture of primary patient material obtained through the OVCARE Tumor bank. All patients with tissue deposited in the OVCARE tumor bank provided written consent for experimental studies including sequencing, IHC characterization, and derivation of long-term cell lines from tissue samples. The OVCARE tumor bank study was approved under University of British Columbia and British Columbia Cancer Agency Research Ethics Board H05-60119 protocol.

All cell lines were subjected to identity testing using STR genotyping (AmpFlSTR Identifiler, Applied Biosystems) at the College of American Pathologist's (CAP) accredited Centre For Translational and Applied Genomics (CTAG) as per manufacturer directives. Only lines with profiles matching public repository records, reported STR [32], and/or original patient tumors (in the case of in-house derived cell lines) were retained for further study.

### Immunohistochemistry and Calculator of Subtype Prediction (COSP)

Cell lines were scraped from culture plates, washed 2× with PBS and pelleted. Cell pellets were re-suspended in ∼500 μl 10% Neutral Buffered Formalin (NBF) and allowed to fix overnight. Cells were pelleted again and re-suspended in a Histo-gel (Thermo-Fisher) plug prior to embedding in paraffin. A tissue microarray (TMA) was constructed as previously described [4] taking 3×2 mm cores from the cell line plugs. Immunohistochemistry (IHC) was performed on 4 μm sections on a Ventana Discovery XT system as previously described [2], [34], refer to table S2 for details of antibodies used. Histotype prediction was done using the Calculator of Subtype Prediction (COSP) [2] in tumor bank mode. Tumour bank mode was chosen due to the nature of the fixed cell lines and the controlled fixation period similar to the tumor bank process on which this predictor was trained. Scoring criteria for IHC was done visually and followed the exact guidelines proposed in the original COSP paper [2]. IHC for mismatch repair (MMR) proteins (Table S5) was performed as described in [35], a complete absence of staining for any given MMR protein resulted in a score of 0 (negative), and is presumed to result in MMR deficiency.

### mRNA transcripts

RNA was extracted from cell lines using Qiazol-miRNeasy kit (Qiagen) protocol and from primary tumors, 12 randomly selected from each histotype, using the miRNeasy FFPE kit (Qiagen). All RNA transcript levels were measured using the NanoString nCounter system [36] and data normalized with nSolver software v1.1 (NanoString Inc.) using endogenous control genes (*ACTB, SDHA, RPL19, POLR1B, PGK1*) as per manufacturers directives. In the case of *TFF3* mRNA levels we considered any sample with detectable transcripts to be positive and substituted a score of “1” in place of TFF3 IHC when using COSP. The detection threshold (DT) for mRNA was considered to be the maximum count from spike-in negative control probes (across all cell line samples) plus 2 standard deviations. Statistical tests were calculated using GraphPad Prism v6.0c software.

### Mutation Testing and Genomic Analysis

Genomic DNA was extracted using standard methods (Gentra Puregene kit; Qiagen). Regions encompassing mutations of known significance (Cancer hotspots) were Sanger sequenced using M13-tagged primers. Sequencing of *ARID1A* was done through a combination of custom hybrid capture and transcriptome sequencing on an Illumina GAII next generation sequencing (NGS) system as described previously [16], [37]. Associated raw data is deposited in the NCBI Sequence Read Archive under BioProjects PRJNA209481, PRJNA209482, and PRJNA209484. All noted variants were either verified by Sanger sequencing or considered validated if recorded in the Cancer Cell Line Encyclopedia (CCLE) [38] and/or the COSMIC database [39]. Expressed re-arrangements were predicted from transcriptome sequencing data for CCC cell lines TOV21G, JHOC-5, JHOC-7, JHOC-9, and RMG-2 using deFuse [40] (Table S3).

### Copy Number Analysis

DNA copy number was inferred from Affymetrix SNP 6.0 genome-wide microarrays. Arrays were run as per manufacturers directives and copy number ratio generated from an unpaired reference. Detection of copy number changed regions was done using a segmentation algorithm. All analysis and visualization was executed with Partek Genomics Suite 6.6, raw data is available from NCBI GEO [Accession GSE48351].

## Results

### Histotype by COSP in ovarian cancer cell lines

**Figure 1:**
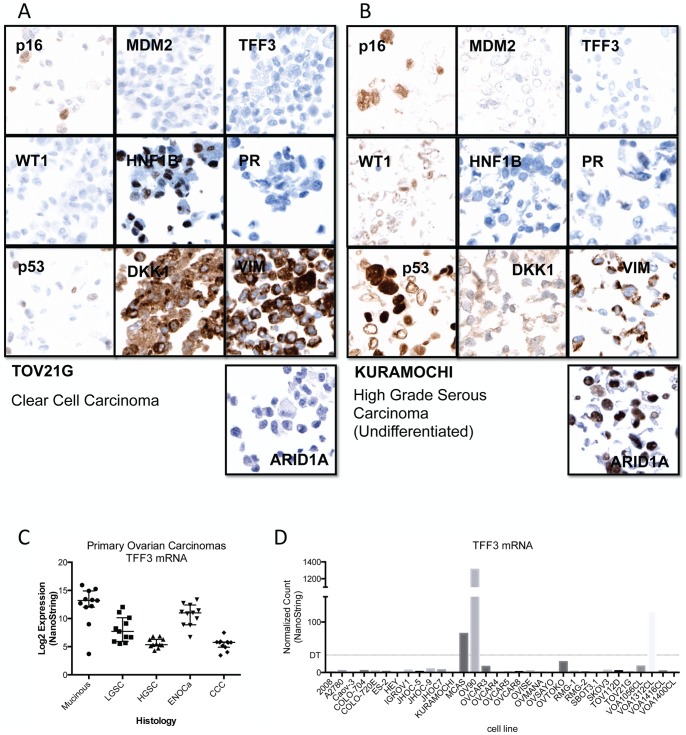
Prediction of histotype was in part based on the COSP algorithm using 9 IHC markers [2]. (A–B) representative IHC from a typical high-grade serous ovarian carcinoma cell line, Kuramochi, and a clear cell carcinoma cell line, TOV21G. In addition to the 9-marker COSP panel, IHC for ARID1A (BAF250a) is also shown as a mutation surrogate. (C) *TFF3* mRNA expression from 60 ovarian cancer samples (12 of each histotype). As noted previously high expression is most prevalent in MUC, followed by ENOCa and LGSC [2], [4]. Expression in our pilot cohort suggests the highest levels of TFF3 in MUC, which was significantly higher than all other groups (Tukey's adjusted p<0.01); no other pairwise comparisons had p<0.05. (D) *TFF3* mRNA detected in ovarian cancer cell lines was used in place of an IHC score as the secreted TFF3 was considered a poor biomarker for cell culture conditions. Any cell line with measurable *TFF3* mRNA above the NanoString detection threshold (see methods) was considered positive (score of 1 for use in the COSP algorithm).

Ovarian cancer cell lines grown in culture do not exhibit the histological phenotypes that are useful for classification into the major disease types. Our group has described a large number of immunohistochemical biomarkers that show specific profiles across these histotypes [4], [41]–[43]. A core panel of 9 IHC markers combined with a predictive algorithm, the Calculator for Ovarian Subtype Prediction (COSP), can be used to reliably distinguish between types [2]. We have previously demonstrated a high level of concordance between our predictive immune-classifier and consensus expert gynecopathological review [2], . Initially, we applied this panel (Fig 1A–B), and the COSP predictive algorithm, to 32 ovarian cancer cell lines of ambiguous histotype to establish if cell lines retained representative characteristics sufficient to classify cell lines to their true disease origins and allow for type-specific ovarian cancer model development. The TFF3 IHC marker, which is normally strongly associated with the mucinous type and seen at moderate frequency in ENOCa and LGSC [2], was negative across all samples (Table S4), suggesting this secreted factor, if expressed at all, may be expelled quickly from the cells and washed away in media. Consequently, TFF3 IHC may not be a reliable biomarker measurement for use with cultured cells. However, the prevalence of *TFF3* mRNA in primary samples appeared similar to that reported by IHC [2], [44], with consistently higher expression in mucinous carcinomas (p<0.01; Fig. 1C). We therefore substituted detectable *TFF3* mRNA for IHC and scored any cell line with detectable mRNA as “1” in our COSP algorithm (Fig. 1D and Table 2).

**Table 2:** Validation of the histotype of commonly used ovarian carcinoma cell lines using immunohistochemistry based prediction via COSP and mutational profiling.

Cell Line	Reported Histotype	COSP Markers									COSP Prediction (Clinical)				Non-COSP Markers	DNA Mutational Profile		Validated Cell Line HistoType
		p16 (CDKN2A)	MDM2	TFF3 [mRNA]	p53	VIMENTIN	WT1	HNF1B	PR	DKK1	CCC	ENOCa	HGSC	MUC	ARID1A (BAF250A)	TP53	Other*	
**JHOC-5**	CCOC	1	0	0	1	1	0	1	0	0	85	13	2	0	1b	nc	none detected	**Clear Cell Carcinoma (CCC**)
**JHOC-7**	CCOC	1	1	0	1	1	0	1	0	0	99	1	0	0	1b	nc	*PIK3CA*	
**JHOC-9**	CCOC	1	0	0	1	1	0	1	0	0	85	13	2	0	0a	nc	*PIK3CA/ARID1A*	
**RMG-2**	CCOC	0	1	0	1	1	0	1	0	1	97	3	0	0	0a	nc	*PPP2R1A/ARID1A*	
**TOV21G**	CCOC	0	0	0	1	1	0	1	0	1	55	41	4	0	0a	nc	*KRAS/PTEN/PIK3CA/ARID1A*	
**OVTOKO**	CCOC	1	1	0	1	1	0	1	0	0	99	1	0	0	0b	nc	none detected	
**OVMANA**	CCOC	1	1	0	1	1	0	1	0	0	99	1	0	0	0a	nc	*PIK3CA/ARID1A*	
**A2780**	Adenocarcinoma	0	0	0	1	1	0	0	0	1	0	94	6	0	0a	nc	*PTEN/ARID1A*	**Endometrioid Carcinoma (ENOCa**)
**IGROV1**	Mixed	1	0	0	1	1	0	1	0	1	17	82	1	0	0a	p.Y126C (het)	*ARID1A/PTEN*	
**TOV112D**	ENOCa	0	0	0	2	1	0	0	0	1	0	38	62	0	1b	p.R175H (Hm)	*CTNNB1*	
**2008**	ENOCa	0	1	0	2	0	0	1	0	0	98	0	2	0	0	c.572_574 del CTC (het)/c.673-1 G>T (het, splice site)	none detected	Atypical Non-Serous [CCC/ENOCa] cell lines**
**OVISE**	CCOC	0	0	0	1	0	0	1	0	1	20	54	25	0	0a	nc	*ARID1A*	
**ES-2**	CCOC	1	0	0	1	1	0	0	0	1	0	100	0	0	1b	p.S241F (het)	*BRAF*	
**SKOV3**	adenocarcinoma	0	0	0	0	0	0	1	1	1	0	0	100	0	1b	nc	*PIK3CA/ARID1A*	
**OVSAYO**	CCOC	0	0	0	2	0	1	0	0	1	0	0	100	0	1	R249M (Hm)	none detected	**High Grade Serous Ovarian Carcinoma (HGSC)**
**CAOV3**	Adenocarcinoma	1	0	0	0	0	0	0	0	1	0	0	100	0	1b	p.Q136* (Hm)	none detected	
**Kuramochi**	Undifferentiated	1	1	0	2	1	1	0	0	0	0	3	97	0	1	p.D281Y (Hm)	none detected	
**OVCAR-3**	Adenocarcinoma	1	0	0	2	0	1	0	0	0	0	0	100	0	1b	p.R248Q (Hm)	none detected	
**OVCAR-4**	Serous Adenocarc.	0	0	0	0	1	0	0	0	1	0	0	100	0	1b	p.L130V (Hm)	none detected	
**OVCAR-5**	Adenocarcinoma	0	0	0	0	0	0	0	0	0	0	0	100	0	1b	nc	*KRAS*	
**OVCAR-8**	Adenocarcinoma	0	0	0	2	1	0	0	0	1	0	38	62	0	1b	p.Y126_splice (Hm)	none detected	
**COLO-720E**	carcinoma	1	1	0	2	0	0	0	0	0	0	20	80	0	1	p.A138V (het)/c.1118delA (het)	*PTEN*	
**COLO-704**	carcinoma	1	0	0	0	0	0	0	0	0	0	0	100	0	1	c.1146delA (het)	*PTEN*	
**Hey**	carcinoma	0	1	0	2	1	0	0	0	0	0	10	90	0	1	nc	*KRAS*	
**VOA1400_CL**	HGSC primary tumour	0	0	0	0	1	1	0	0	1	0	0	100	0	1	E198* (het)	none detected	
**VOA1416_CL**	HGSC ascites	0	0	0	0	1	1	0	0	1	0	0	100	0	1	nc	none detected	
**VOA1072_CL**	HGSC primary tumour	0	0	0	2	0	0	0	0	0	0	0	100	0	1	R248Q (Hm)	none detected	
**VOA1312_CL**	LGSC ascites	0	1	1	1	1	0	0	0	1	0	100	0	0	1	nc	*KRAS*	**Low-Grade Serous Carcinoma (LGSC)**
**VOA1056_CL**	LGSC primary tumour	0	0	0	1	1	0	0	0	0	0	39	61	0	1	nc	*NRAS*	
**MCAS**	mucinous carcinoma	0	0	1	2	0	0	0	0	1	0	0	0	100	1b	127bp del (Hm, Ex 4)	*KRAS*	**mucinous carcinoma**
**RMG-1**	CCOC	0	0	0	0	0	0	1	0	1	22	0	76	3	1	nc	none detected	**unclassified**
**OV90**	Adenocarcinoma	1	1	1	2	0	0	1	1	0	0	53	47	0	1b	p.S215R (Hm)	none detected	

COSP and AIRD1A markers were scored as positive (1) or negative (0), except for p53: null mutation (0), wildtype (1), mutated (2).

ARID1A IHC: a – corresponding ARID1A nonsense or frameshift mutation detected, b – no ARID1A mutation detected in sequencing data (if no letter code, sequence information was unavailable)

COSP algorithm can be found at http://www.gpec.ubc.ca/index.php?content=papers/ovcasubtype.php

TP53 mutations are noted as heterozygous (het) or Homozygous/Hemizygous (Hm)

* Sequencing of BRAF, KRAS, ERBB2, NRAS, CTNNB1, EGFR, PTEN, PIK3CA, PPP2R1A, DICER1 and ARID1A

Many previously described CCC lines showed features characteristic of their expected origins. In addition to the COSP 9-marker panel, we added IHC for ARID1A (BAF250a). Given the strong negative association of mutation status and detectable protein expression [16] we considered this assay as a surrogate mutation test useful in segregating endometriosis associated ovarian cancer from other subtypes, most notably high-grade serous, as *ARID1A* mutations appear to be exceedingly rare in this subtype [16], [45].

### Mutational Profiles: Clear cell specific molecular features

We next tested cell lines for mutations in common ovarian cancer associated genes (Table 2 and Table S5). As some of the cell lines we tested are also part of a larger Cancer Cell Line Encyclopedia (CCLE) repository data set [38], we cross-validated our mutation testing with this database as well as the COSMIC database [39]. We focused on regions of known significance in common cancer genes including hotspots in *BRAF, KRAS, NRAS, ERBB2, EGFR, CTNNB1, PIK3CA, PPP2R1A* and *DICER1*. All coding exons of *TP53* were verified in all cell lines. *ARID1A* mutations were tested using a custom NGS gene hybrid capture strategy [37] in RMG-1, RMG-2, JHOC-5, JHOC-7, JHOC-9, TOV21G, and ES-2; for all other cell lines we used *ARID1A* data from COSMIC and CCLE in addition to IHC as an *ARID1A* mutation-testing surrogate (Table 2).

As with our IHC data most CCC lines maintained a profile consistent with the CCC histotype including mutations in *PIK3CA* and *ARID1A*. Further, loss of ARID1A expression, demonstrated by IHC, showed good concordance with presence of known truncating mutations, as noted for primary tumor specimens [16]. As expected IHC for p53 correlated well with occurrence of mutations. For cell lines with a recorded mutation (at time of submission) in either CCLE or COSMIC all detected mutations matched repository records, except for a homozygous/hemizygous 127-bp deletion of *TP53* detected in MCAS. We presume that the 127-bp deletion in MCAS (at the end of exon 4; Fig. S1) may have been overlooked in the CCLE exon sequencing strategy as in our experience false negatives are prevalent in NGS datasets. Overall, the addition of mutation data was particularly helpful in supporting initial classification from COSP (Table 2).

We took note that a number of cell lines often used as high-grade serous models or generically as “ovarian carcinoma” had both *ARID1A* mutations and immuno-profiles consistent with the endometrioid type, the third most common type accounting for less than 10% of ovarian carcinomas [3], [4]. Along with the immuno-classification the presence of an *ARID1A* mutation provided compelling evidence of a non-HGSC origin. The incidence of *TP53* and *ARID1A* mutation was near mutually exclusive with the exceptions of IGROV1 and 2008. IGROV1 carries two frame shift mutations in *ARID1A* (p.M274fs/p.G1847fs) and a mutation of unknown significance in *TP53* (p.Y126C (het)), though p53 expression by IHC appeared normal. The 2008 cell line had undetectable ARID1A, suggesting loss of function, and also carried two *TP53* mutations (c.572_574 delCTC (het)/c.673-1 G>T (het, splice site)) and corresponding p53 IHC overexpression. These atypical combinations of mutation could plausibly be explained by a propensity to accumulate mutations in cell lines with DNA mismatch repair (MMR) deficiencies, as has been reported for IGROV1, SKOV3, and A2780 [46], [47]. We validated MMR pathway protein expression with IHC for MLH-1, PMS-2, MSH-2, and MSH-6 (Table S5) and observed loss of two or more MMR proteins in IGROV1, SKOV3, A2780 TOV21G, COLO-704 and COLO-720E; no MMR protein deficiency was noted in the 2008 cell line.

### Copy Number profiles of clear cell lines

As our primary objective was to describe CCC cell lines we generated copy number profiles of bona-fide CCC cell lines using Affymetrix SNP6.0 microarrays. Consistent with previous reports using primary tumor samples, CCC lines showed a moderate degree of copy number abnormalities, suggesting a genome that has undergone some degree of genomic instability.

**Figure 2:**
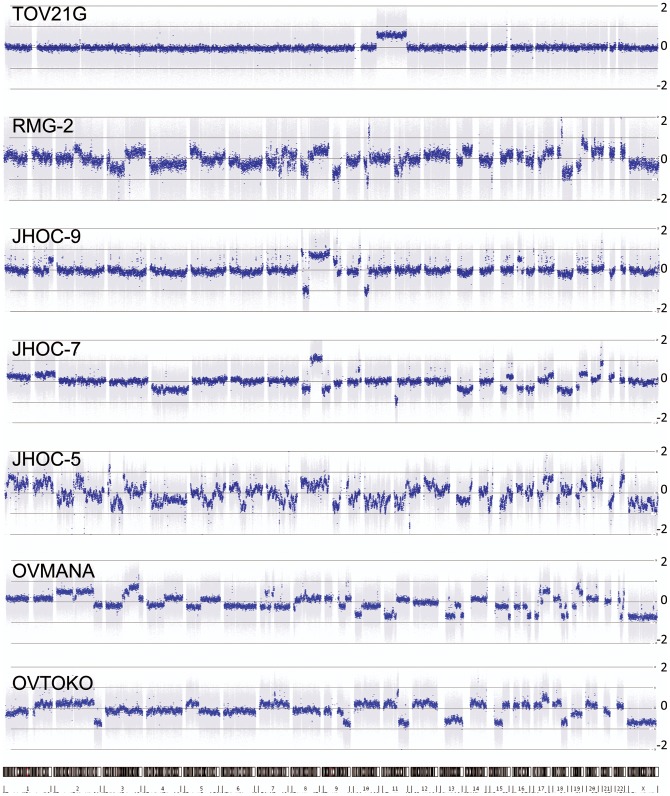
Genome-wide copy number profiles of bona-fide ovarian CCC cell lines. A large range of copy number changes are seen including typical Chr8 gains and Chr17 gains surrounding the CCC biomarker *HNF1B* gene, see also Table 3.

A limited number of literature reports have highlighted genes with mutations, overexpression and/or amplification amongst primary CCC, some with a relationship to survival or advanced disease [4], [16], [45], [48]–[59]. As we observed in mutation profiles, our bona-fide CCC cell line panel was representative of clear-cell associated copy number changes (Figure 2, Table 3). Most showed modest copy number gains for *HNF1B* (5/7) and *MET (4/7)*, including one with high-level amplification (JHOC-5), similar to previous reports for CCC tumors [53], [56], [57]. Although 3/7 CCC lines showed copy number gain of *ERBB2*, in all cases the amplicon segment also encompassed the nearby CCC biomarker *HNF1B*, and none were positive for HER2 protein expression by IHC (not shown). Copy number loss around *TP53* was observed only in a single CCC cell line (OVMANA; heterozygous loss) and, as noted above, all CCC lines appeared to have a normal-like expression pattern for p53 (IHC score 1).

**Table 3:** Copy number changes across putative CCC oncogenes, tumor suppressors, and biomarkers.

Segment Copy Number	JHOC-5	JHOC-7	JHOC-9	OVMANA	OVTOKO	RMG-2	TOV21G	References
**ARID1A**	3.422	2.397	NC	2.314	NC	NC	NC	[16], [45]
**ERBB2**	3.061	NC	NC	NC	2.514	2.382	NC	[52]
**HNF1B**	3.061	3.109	3.533	NC	2.514	2.382	NC	[53]
**MAP1LC3A**	NC	NC	NC	NC	NC	NC	NC	[54]
**MET**	8.465	NC	NC	3.451	2.346	2.667	NC	[53], [56], [57]
**PIK3CA**	0.971	1.222	NC	NC	NC	6.482	NC	[48], [49]
**PPM1D**	3.157	2.329	NC	3.004	3.009	2.382	NC	[50], [51]
**STAT3**	3.314	3.142	NC	NC	2.522	2.382	NC	[53]
**TP53**	NC	NC	NC	1.320	2.410	NC	NC	[4]
**YAP1**	NC	NC	NC	NC	1.264	NC	NC	[59]
**ZNF217**	2.893	5.897	4.717	3.412	3.648	2.589	NC	[55], [58]
**CDKN2A**	0.163	NC	NC	NC	0.246	1.244	NC	[58]
**CDKN2B**	0.454	NC	NC	NC	0.602	1.244	NC	[58]

NC  =  no change in copy number was detected.

### Transcriptome profile of clear cell lines

**Figure 3:**
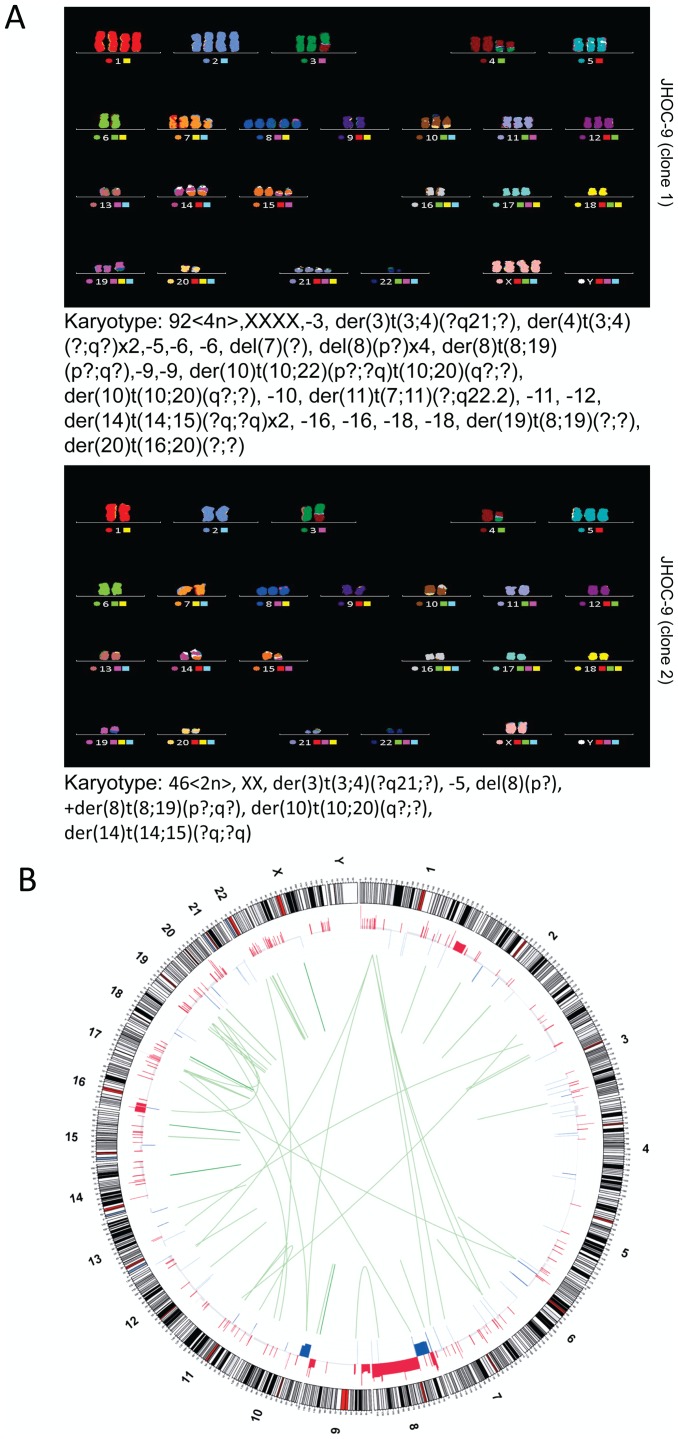
Genomic structure of CCC cell line JHOC-9. (A) 24 color FISH analysis suggested the presence of two dominant clones; one near-diploid and one near-tetraploid in the JHOC-9 CCC cell line. A number of translocations and rearrangements can be seen in each representative clone. The complex karyotype of each dominant clone is noted below the corresponding 24-colour FISH results. Not all derivative chromosomes were identifiable resulting in a large number of ambiguous translocations and fragments (denoted by question marks in the karyotype notations). (B) Circos plot of RNAseq data and deFuse analysis depicting expressed genomic rearrangements in the JHOC-9 cell line. Translocations seen in the 24-color FISH profile are also visible as expressed transcripts including t(8;19) observed in both 2N and 4N dominant clones. No recurrent translocations were seen across our series (see also Table S3).

As with other ovarian carcinoma types, recurrent translocations amongst CCC have not been described, though only a minimal number of studies have been undertaken [16]. Our transcriptome sequencing data on RMG-1, RMG-2, JHOC-5, JHOC-7, JHOC-9, TOV21G, and ES-2 suggests recurrent expressed rearrangements are at least rare and were not detected amongst these cell lines. A moderate number of expressed intra- and inter-chromosomal rearrangements were detectable (Table S4), though all were unique to each respective cell line; some were visible by multi-colored FISH (Figure 3). Both expressed and non-expressed translocations resulting in gene gain/loss of function or promoter exchange may serve to influence pathway activation, and overall expression, profiles of CCC. A systematic analysis was considered beyond the scope of this study and there is currently an absence of an equivalent knowledge base derived from primary CCC tumors for comparison.

## Discussion and Conclusions

As our initial goal was identification of bonafide CCC cell lines, we are pleased to report that the majority of reported CCC lines were representative of the primary tumors' molecular and pathological phenotype. Our immuno-classification scheme, COSP, predicted most to be CCC and our own mutation data, as well as that from COSMIC and CCLE, suggested loss of function *ARID1A* mutations were prevalent in these cell lines. Although three CCC lines did not have identifiable *ARID1A* mutations, only JHOC-5 cells appeared to have both wild-type sequence and detectable protein expression. The number of ARID1A “normal” CCC lines is lower than might be expected given the frequency of *ARID1A* mutations (and negative IHC) observed in primary CCC [16], [45] and may indicate some preferential selection for *ARID1A* null CCC lines to adapt to *in vitro* culture. However, given the small sample size it may well be a chance occurrence and does not appear to be significant. Other detected mutations (*PIK3CA*, *PTEN*, *KRAS*, *PPP2R1A*) are all consistent with varying frequencies in CCC. *TP53* mutations are notably absent in all of our validated CCC cell lines, as a de-facto defining characteristic, and only a single CCC line had heterozygous copy number loss though still retained normal-like p53 IHC.

Both CCC and ENOCa appear to arise in a background of endometriosis. Atypical endometriosis adjacent to, or contiguous with, either histotype is not unusual for either CCC or ENOCa [16], [60], [61]. Co-occurrence (sometimes contiguous) of both CCC and ENOCa histologies in a mixed-cell type tumor has been reported [62] (and Dr. Blake Gilks, personal communication). Mutational profiles including *ARID1A* and *PIK3CA*, are common to both types, overall supporting a related origin and similar route to transformation [16], [45], [48], [49]. We found that both ES2 and OVISE cell lines, reportedly derived from CCC, largely resembled the immuno-profiles of ENOCa. Conversely the 2008 cell line, reportedly derived from serous carcinoma [63], though often referred to as ENOCa [64], appeared more CCC-like from COSP alone. The 2008 line did show mutant p53 staining and has two confirmed *TP53* mutations, atypical for true CCC. IHC was negative for ARID1A, supporting a non-serous origin. We favored an assignment of ENOCa base largely on the *TP53* mutation though note that this cell line is quite atypical as it may carry loss of function changes for ARID1A, mutation of *TP53*, and is positive for the CCC biomarker HNF1B. Arguably errors in cell line histotype reports may be explained simply by historically poor reproducibility in cell type assignment, though it is not unforeseeable that the biological relationship between CCC and ENOCa could be influencing these phenotypes. Given the high degree of overlap between the mutational characteristics of CCC and ENOCa, our panel was not able to further segregate or clarify this apparent confusion. SKOV3 is another unique case as it's immuno-phenotype most closely resembles HGSC, yet it caries a truncating mutation for *ARID1A*, a mutation that has not been observed in HGSC despite widespread testing [16], [45]. Previous studies with SKOV3 have pointed to a clear cell-like histology when grown as xenograft [64] and this may also favor an endometriosis-associated ovarian cancer diagnosis as does the presence of a *PIK3CA* mutation. Finally, the TOV112D cell line also presents as an exception with a moderately strong prediction of HGSC immuno-phenotype. In spite of this finding we suggest this line is representative of *TP53* mutant ENOCa, based on the presence of an ENOCa characteristic *CTNNB1* mutation, pathological review of the primary tumor material in the originating laboratory and expression profiling experiments supporting this conclusion [65]. We propose that these atypical CCC/ENOCa may be useful in exploration of some common endometriosis-associated ovarian cancer biology though care should be undertaken to allow proper interpretation of the results.

Unfortunately our COSP tool is unable to differentiate LGSC. Based on expert re-review of primary material we are aware of two cell lines derived from LGSC primary tumors. We therefore confidently favor this classification for VOA1056_CL and VOA1312_CL despite predictions of HGSC or ENOCa obtained from COSP. The VOA1056_CL line carries a Ras-pathway mutation as might be expected of an LGSC tumor, however this is an NRAS Q61R activating mutation. Activating NRAS mutations were recently described in LGSC at the 2012 AACR annual meeting [66] however, this represent the first validated report of an *NRAS* mutant LGSC tumor and the first validated LGSC derived cell line carrying this mutation. The COSMIC database suggests the cell lines LK-1 (G12D; defined as ovarian carcinoma, type not specified) and TYK-nu (G12D and Q61K; defined as ovarian “serous carcinoma”) also carry activating *NRAS* mutations, however we were unable to source these cell lines to confirm/reject their histological identity. In the cases of LGSC cell lines derived in-house, mutations of *TP53* were not observed, consistent with IHC based literature reports suggesting this is a major molecular discriminator between HGSC and LGSC [67].

Finally only a single cell line in our collection was reported to be of mucinous carcinoma origin. The mutation profile of this cell line is consistent with this diagnosis, including a 127-bp *TP53* homozygous deletion, overexpression by IHC, and *KRAS* G12V mutation.

Cell line records for epithelial ovarian carcinoma have recently come into question with a number contaminated and redundant cell lines acknowledged in a recent study [32]. Most notably 2008 (aka. ov2008) was reported to be frequently contaminated with, or a mislabeled version of, the HPV-positive ME-180 cell line (ATCC HTB-33), the “true” HPV-negative 2008 line defined in the report from Korch et al. [32], is the one used in our study. Maintaining appropriate records, testing and, most importantly, re-testing identity of cell lines in each individual lab's stocks should be paramount even if cell lines are obtained directly from repositories. Here we report only on cell lines that matched the originating repository STR DNA profile or the STR profile of their originating primary tumors (in the case of in-house derived lines). Despite our own best efforts our exercise did yield the discovery of 3 cell lines in our own stocks that were either mislabeled or contaminated, including our original stock of the 2008 cell line noted above. All contaminated lines have since been discarded/replaced. It should be noted that none of our assays were designed or tested to discriminate ovarian from non-ovarian malignancies, and although STR analysis would have eliminated any obviously male cancers (through detection of Chr Y markers), some level of accuracy in repository reported origin of “ovarian” must be assumed. In the case of the more atypical cell lines it is possible these may be of non-ovarian origin, e.g. endometrial carcinomas or other peritoneal cancers of unknown primary, we are currently unable to assess this idea. Further, our analysis may be confounded by dominant expansion of rare tumor sub-clones [68], acquisition of spontaneous mutations during culturing, and MMR deficiency (whether acquired or present in the originating primary tumor). MMR deficiencies have been reported to be prevalent in endometriosis-associated ovarian cancers (CCC and ENOCa) [69]–[72] and the potential acquisition of mutations as a result of MMR deficiency may influence some of the more ambiguous biomarker phenotypes within this group, as well as observed atypical mutation patterns. We noted MMR deficiencies in the non-serous lines TOV21G, SKOV3, A2780, and IGROV1 as well as the HGSC cell lines COLO-704 and COLO-720E (Table S5). MMR-protein deficiencies were not observed in our in-house derived LGSC cell lines (or their corresponding primary tumors) or in the mucinous carcinoma line MCAS.

In the spectrum of ovarian carcinomas, recent evidence strongly supports diagnosis and treatment of the five major histotypes of carcinomas as distinct diseases. Cancer cell lines provide an important intermediate tool for clinically relevant translational science, allowing genomic manipulation and cell biology studies beyond what can be reasonably achieved in clinical trials or animal models of cancer. In order to develop appropriate treatments, translational researchers need to use model systems appropriate to each ovarian carcinoma type. Unfortunately, historical records of ovarian cancer cell lines have rarely included information on histological origin [32], [64]; this is further hampered by a historical lack of reproducibility in histological diagnosis [73]–[75]. Histopathological reproducibility is steadily improving as recognition of the five major histotypes as unique disease entities becomes more widespread [4], [7], [8], [15], [22], [44], [76], [77], histology and grading criteria become unified [78]–[81], and objective biomarker based tools to delineate histotypes are developed [2], [4], [41], [44]. However, cell lines lack morphological features that are recognizable in culture and development of new, well-defined, cell lines is laborious with poor long-term success rates. Assigning histotype to readily available and well-used cell lines will undoubtedly lead to better interpretation of new data and re-interpretation of already published findings.

## Supporting Information

Figure S1 **127bp homozygous (or hemizygous) deletion affecting** *TP53* **exon 4 in the MCAS mucinous carcinoma cell line.** This mutation was apparent by Sanger sequencing though not annotated in the CCLE database. Coding bases are annotated in upper case.
(PDF)

Table S1 **Cell Lines & Sources.**
(PDF)

Table S2 **Antibodies and Dilutions.**
(PDF)

Table S3 **deFuse predicted expressed re-arrangements from transcriptome sequencing data.**
(XLS)

Table S4 **Mutations Found In Ovarian Carcinoma Cell Lines.**
(PDF)

Table S5 **Mismatch Repair IHC.**
(PDF)
