# Correction: Type-Specific Cell Line Models for Type-Specific Ovarian Cancer Research

**DOI:** 10.1371/annotation/ffcaf179-872f-470b-8bb6-f06d8ba6d03a

**Published:** 2013-09-27

**Authors:** Michael S. Anglesio, Kimberly C. Wiegand, Nataliya Melnyk, Christine Chow, Clara Salamanca, Leah M. Prentice, Janine Senz, Winnie Yang, Monique A. Spillman, Dawn R. Cochrane, Karey Shumansky, Sohrab P. Shah, Steve E. Kalloger, David G. Huntsman

There were errors in the Funding section. The correct version of the section is available below.

Support for this project was provided by the Canadian Institutes for Health Research (CIHR) Emerging Team Grant: Personalized siRNA-Based Nanomedicines (FRN: 111627), the VGH and UBC Hospital Foundation, and the Gray Family Ovarian Clear Cell Carcinoma Research Resource through the BC Cancer Foundation to the Ovarian Cancer Research Team of BC (OVCARE; http://www.ovcare.ca). The funders had no role in study design, data collection and analysis, decision to publish, or preparation of the manuscript.

