# Correction: Type-Specific Cell Line Models for Type-Specific Ovarian Cancer Research

**DOI:** 10.1371/annotation/856f0890-9d85-4719-8e54-c27530ac94f4

**Published:** 2013-10-09

**Authors:** Michael S. Anglesio, Kimberly C. Wiegand, Nataliya Melnyk, Christine Chow, Clara Salamanca, Leah M. Prentice, Janine Senz, Winnie Yang, Monique A. Spillman, Dawn R. Cochrane, Karey Shumansky, Sohrab P. Shah, Steve E. Kalloger, David G. Huntsman

There were formatting errors in Table 2. A correct version of the table is available here: http://plosone.org/corrections/pone.0072162.t002.cn.tif

