# Supplementary material for: Type-Specific Cell Line Models for Type-Specific Ovarian Cancer Research
Source: PLoS One. 2013 Sep 4;8(9):e72162. doi: 10.1371/journal.pone.0072162 (PMC3762837; doi:10.1371/journal.pone.0072162)
Supplement: Figure S1 — 127bp homozygous (or hemizygous) deletion affecting TP53 exon 4 in the MCAS mucinous carcinoma cell line. This mutation was apparent by Sanger sequencing though not annotated in the CCLE database. Coding bases are annotated in upper case. (PDF) [file pone.0072162.s001.pdf]

Supplemental Figure S1

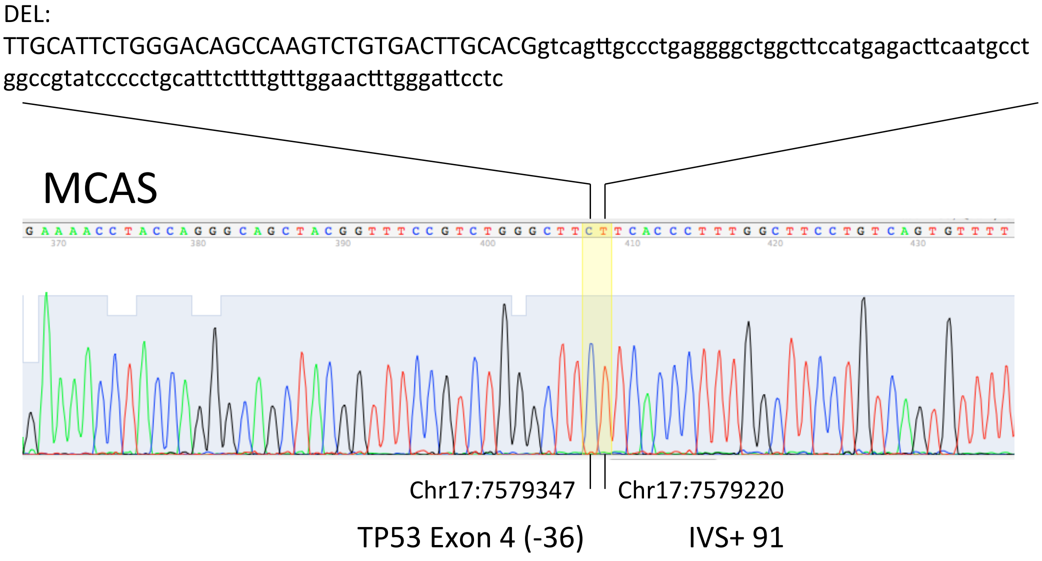

Supplemental Fig S1: 127bp homozygous (or hemizygous) deletion affecting *TP53* exon 4 in the MCAS mucinous carcinoma cell line. This mutation was apparent by Sanger sequencing though not annotated in the CCLE database. Coding bases are annotated in upper case.
