# Supplementary material for: Type-Specific Cell Line Models for Type-Specific Ovarian Cancer Research
Source: PLoS One. 2013 Sep 4;8(9):e72162. doi: 10.1371/journal.pone.0072162 (PMC3762837; doi:10.1371/journal.pone.0072162)
Supplement: Table S1 — Cell Lines & Sources. (PDF) [file pone.0072162.s002.pdf]

Supplemental Table S1: Cell Lines & Sources

| Cell Line | Reported Histotype | Media | SOURCE | STR Reference |
|---|---|---|---|---|
| 2008 | ENOCa | RPMI/10% FBS | UC Denver (via M. Spillman; no public repository source) | Korch et al. |
| A2780 | Adenocarcinoma | RPMI/10% FBS | ECACC (via I. Campbell) | ATCC |
| CAOV3 | Adenocarcinoma | 199/105 5% FBS | ATCC | ATCC |
| COLO-704 | Carcinoma | RPMI/10% FBS | UC Denver cell bank (via M. Spillman) | DSMZ STR Profiler/ Korch et al. |
| COLO-720E | Carcinoma | RPMI/10% FBS | UC Denver cell bank (via M. Spillman) | DSMZ STR Profiler/ Korch et al. |
| ES-2 | CCC | RPMI/10% FBS | ATCC | ATCC |
| HEY | Carcinoma | RPMI/10% FBS | ATCC (via UC Denver/M. Spillman) | DSMZ STR Profiler/ Korch et al. |
| IGROV1 | Cystoadenocarcinoma | McCoy 5A 5% FBS | NCI cell bank | Cell Miner |
| JHOC-5 | CCC | RPMI/10% FBS | RIKEN Cell Bank | RIKEN (DSMZ STR Profiler) |
| JHOC-7 | CCC | RPMI/10% FBS | RIKEN Cell Bank | RIKEN (DSMZ STR Profiler) |
| JHOC-9 | CCC | RPMI/10% FBS | RIKEN Cell Bank | RIKEN (DSMZ STR Profiler) |
| Kuramochi | Undifferentiated | RPMI/10% FBS | HSRRB Cell Bank | HSRRB |
| MCAS | Mucinous | RPMI/10% FBS | JCRB Cell Bank (via UC Denver/M. Spillman) | DSMZ STR Profiler/ Korch et al. |
| OV90 | Adenocarcinoma | 199/105 5% FBS | ATCC | ATCC |
| OVCAR-3 | Adenocarcinoma | 199/105 5% FBS | T.C. Hamilton (via N. Auersperg) | ATCC/Cell Miner |
| OVCAR-4 | Adenocarcinoma | 199/105 5% FBS | T.C. Hamilton (via N. Auersperg) | ATCC/Cell Miner |
| OVCAR-5 | Adenocarcinoma | 199/105 5% FBS | T.C. Hamilton (via N. Auersperg) | ATCC/Cell Miner |
| OVCAR-8 | Adenocarcinoma | 199/105 5% FBS | NCI cell bank (via P. Olive) | ATCC/Cell Miner |
| OVISE | CCC | RPMI/10% FBS | JCRB Cell Bank (via J.Brenton) | JCRB |
| OVMANA | CCC | RPMI/10% FBS | JCRB Cell Bank (via J.Brenton) | JCRB |
| OVSAYO | adenocarcinoma | RPMI/10% FBS | JCRB Cell Bank (via J.Brenton) | JCRB |
| OVTOKO | CCC | RPMI/10% FBS | JCRB Cell Bank (via J.Brenton) | JCRB |
| RMG-1 | CCC | RPMI/10% FBS | HSRRB Cell Bank | HSRRB |
| RMG-2 | CCC | RPMI/10% FBS | HSRRB Cell Bank | HSRRB |
| SKOV3 | adenocarcinoma | 199/105 5% FBS | ATCC | ATCC |
| TOV112D | ENOCa | 199/105 5% FBS | ATCC | ATCC |
| TOV21G | CCC | 199/105 5% FBS | ATCC | ATCC |
| VOA1056_CL | LGSC | 199/105 10%FBS | OvCaRe derived (C. Salamanca) | OvCaRe (primary tumour) |
| VOA1072_CL | HGSC | 199/105 10%FBS | OvCaRe derived (C. Salamanca) | OvCaRe (primary tumour) |
| VOA1312_CL | LGSC | 199/105 10%FBS | OvCaRe derived (C. Salamanca) | OvCaRe (primary tumour) |
| VOA1400_CL | HGSC | 199/105 10%FBS | OvCaRe derived (C. Salamanca) | OvCaRe (primary tumour) |
| VOA1416_CL | HGSC | 199/105 10%FBS | OvCaRe derived (C. Salamanca) | OvCaRe (primary tumour) |
