# Supplementary material for: Type-Specific Cell Line Models for Type-Specific Ovarian Cancer Research
Source: PLoS One. 2013 Sep 4;8(9):e72162. doi: 10.1371/journal.pone.0072162 (PMC3762837; doi:10.1371/journal.pone.0072162)
Supplement: Table S2 — Antibodies and Dilutions. (PDF) [file pone.0072162.s003.pdf]

Supplemental Table S2: Antibodies and Dilutions

Antibody	Supplier	Catalogue #	Host	Clone	Staining Platform	Detection System	Antigen Retrieval	1° Antibody	2° Antibody	3° Antibody
**DKK1**	R&D Systems	AF1096	Goat	Polyclonal	Ventana Discovery XT	ChromoMap DAB	Protease 2, 8 min	1:25, 2 hr, no heat	1:500, Jackson unconj. Rb anti-Gt IgG, 32 min	UltraMap anti-Rb HRP, 16 min
**HNF1B**	Sigma-Aldrich	HPA002083	Rabbit	Polyclonal	Ventana Discovery XT	DAB Map	CC1, Standard	1:100, 1 hr, heat	Universal Secondary, 32 min	n/a
**MDM2**	Zymed	18-2403	Mouse	IF2	Ventana Discovery XT	ChromoMap DAB	CC1, Standard	1:50, 1 hr, no heat	UltraMap anti-Ms HRP, 16 min	n/a
**P16**	mtm laboratories	9518	Mouse	E6H4	Ventana Discovery XT	ChromoMap DAB	CC1, Standard	1:2, 2 hr, no heat	UltraMap anti-Ms HRP, 16 min	n/a
**P53**	Dako	M7001	Mouse	DO-7	Ventana Discovery XT	DAB Map	CC1, Standard	1:400, 1 hr, heat	Universal Secondary, 32 min	n/a
**PR**	Ventana	790-2223	Rabbit	1E2	Ventana Discovery XT	DAB Map	CC1, Standard	neat, 8 min, heat	Universal Secondary, 32 min	n/a
**TFF3**	Abnova	H00007033-M01	Mouse	3D9	Ventana Discovery XT	DAB Map	CC2, Standard	1:50, 1 hr, heat	Universal Secondary, 32 min	n/a
**Vimentin**	Zymed	18-0052	Mouse	V9	Ventana Discovery XT	DAB Map	CC2, Mild	1:50, 32 min, heat	Universal Secondary, 32 min	n/a
**ARID1A**	Sigma-Aldrich	HPA005456	Rabbit	Polyclonal	Ventana Discovery XT	DAB Map	CC1, Standard	1:75, 1 hr, heat	Universal Secondary, 32 min	n/a
**WT1**	Dako	M3561	Mouse	6F-H2	Ventana Discovery XT	DAB Map	CC1, Standard	1:50, 1 hr, heat	Universal Secondary, 32 min	n/a
