# Supplementary material for: Type-Specific Cell Line Models for Type-Specific Ovarian Cancer Research
Source: PLoS One. 2013 Sep 4;8(9):e72162. doi: 10.1371/journal.pone.0072162 (PMC3762837; doi:10.1371/journal.pone.0072162)
Supplement: Table S4 — Mutations Found In Ovarian Carcinoma Cell Lines. (PDF) [file pone.0072162.s005.pdf]

Supplemental Table S4: Mutations Found In Ovarian Carcinoma Cell Lines

Cell Line / Mutation tested	Present in CCLE	BRAF V599/V600	KRAS G12/G13	ERBB2 ins. 774-781	NRAS.1 G12/G13	NRAS.2 G60/G61	CTNNB1 32,33,34,35 38,41,45	EGFR.1 868	EGFR.2 740-750	EGFR.3 770	PTEN.1 130	PTEN.2 173	PTEN.3 233	PIK3CA.1 542-546	PIK3CA.2 1047	PPP2R1A	DICER1.1 1705/1709	DICER1. 1818/1813	TP53 mutation	TP53 Mutation TYPE	ARID1A Mutations truncating (NS/Frame) mutations only
**A2780**	*YES*	nc	nc	nc	nc	nc	nc	nc	nc	nc	383_391delA GGGACGAA	nc	nc	nc	nc	nc	nc	nc	nc	nc	p.Q1430* / p.R1721fs
**CAOV3**	*YES*	nc	nc	nc	nc	nc	nc	nc	nc	nc	nc	nc	nc	nc	nc	nc	nc	nc	p.Q136*	Nonsense Mutation	
**ES-2**	*YES*	V600E	nc	nc	nc	nc	nc	nc	nc	nc	nc	nc	nc	nc	nc	nc	nc	nc	p.S241F	Missense Mutation	
**IGROV1**	*YES*	nc	nc	nc	nc	nc	nc	nc	nc	nc	Y155C	nc	nc	nc	nc	nc	nc	nc	p.Y126C	Missense Mutation	p.M274fs / p.G1847fs
**JHOC-5**	*YES*	nc	nc	nc	nc	nc	nc	nc	nc	nc	nc	nc	nc	nc	nc	nc	nc	nc	nc	nc	
**JHOC-7**	*NO*	nc	nc	nc	nc	nc	nc	nc	nc	nc	nc	nc	nc	E542K	nc	nc	nc	nc	nc	nc	
**JHOC-9**	*NO*	nc	nc	nc	nc	nc	nc	nc	nc	nc	nc	nc	nc	E542K(Hm)	nc	nc	nc	nc	nc	nc	p.E1779*
**Kuramochi**	*YES*	nc	nc	nc	nc	nc	nc	nc	nc	nc	nc	nc	nc	nc	nc	nc	nc	nc	p.D281Y	Missense Mutation	
**OV90**	*YES*	nc	nc	nc	nc	nc	nc	nc	nc	nc	nc	nc	nc	nc	nc	nc	nc	nc	p.S215R	Missense Mutation	
**OVCAR-3**	*YES*	nc	nc	nc	nc	nc	nc	nc	nc	nc	nc	nc	nc	nc	nc	nc	nc	nc	p.R248Q	Missense Mutation	
**OVCAR-4**	*YES*	nc	nc	nc	nc	nc	nc	nc	nc	nc	nc	nc	nc	nc	nc	nc	nc	nc	p.L130V	Missense Mutation	
**OVCAR-5**	*NO*	nc	G12V	nc	nc	nc	nc	nc	nc	nc	nc	nc	nc	nc	nc	nc	nc	nc	nc	nc	
**OVCAR-8**	*YES*	nc	nc	W746C	nc	nc	nc	nc	nc	nc	nc	nc	nc	nc	nc	nc	nc	nc	p.Y126_splice	Splice Site SNP	
**OVISE**	*YES*	nc	nc	nc	nc	nc	nc	nc	nc	nc	nc	nc	nc	nc	nc	nc	nc	nc	nc	nc	
**OVMANA**	*YES*	nc	nc	nc	nc	nc	nc	nc	nc	nc	nc	nc	nc	E545V	nc	nc	nc	nc	nc	nc	p.Q1332* / p.S2264*
**OVSAYO**	*NO*	nc	nc	nc	nc	nc	S37F	nc	nc	nc	nc	nc	nc	nc	H1047R	nc	nc	nc	R249M(Hm)	Missense Mutation	
**OVTOKO**	*YES*	nc	nc	nc	nc	nc	nc	nc	nc	nc	nc	nc	nc	nc	nc	nc	nc	nc	nc	nc	
**RMG-1**	*YES*	nc	nc	nc	nc	nc	nc	nc	nc	nc	nc	nc	nc	nc	nc	nc	nc	nc	nc	nc	
**RMG-2**	*NO*	nc	nc	nc	nc	nc	nc	nc	nc	nc	nc	nc	nc	nc	nc	R183W	nc	nc	nc	nc	
**SKOV3**	*YES*	nc	nc	nc	nc	nc	nc	nc	nc	nc	nc	nc	nc	nc	H1047Y	nc	nc	nc	nc	nc	p.Q586*
**TOV112D**	*YES*	nc	nc	nc	nc	nc	S37A	nc	nc	nc	nc	nc	nc	nc	nc	nc	nc	nc	R175H	Missense Mutation	
**TOV21G**	*YES*	nc	G13C	nc	nc	nc	nc	nc	nc	nc	148delG	nc	indel 800del	nc	H1047Y	nc	nc	nc	nc	nc	p.Q548fs / p.N756fs
**VOA1056_CL**	*NO*	nc	nc	nc	nc	Q61R	nc	nc	nc	nc	nc	nc	nc	nc	nc	nc	nc	nc	nc	nc	
**VOA1072_CL**	*NO*	nc	nc	nc	nc	nc	nc	nc	nc	nc	nc	nc	nc	nc	nc	nc	nc	nc	R248Q(Hm)	Missense Mutation	
**VOA1312_CL**	*NO*	nc	G12V	nc	nc	nc	nc	nc	nc	nc	nc	nc	nc	nc	nc	nc	nc	nc	nc	nc	
**VOA1400_CL**	*NO*	nc	nc	nc	nc	nc	nc	nc	nc	nc	nc	nc	nc	nc	nc	nc	nc	nc	E198*	Nonsense Mutation	
**VOA1416_CL**	*NO*	nc	nc	nc	nc	nc	nc	nc	nc	nc	nc	nc	nc	nc	nc	nc	nc	nc	nc	nc	
**2008**	*NO*	nc	nc	nc	nc	nc	nc	nc	nc	nc	nc	nc	nc	nc	nc	nc	nc	nc	c.572_574 delCTC (het) / c.673-1 G>T (het, splice site)	Nonsense Mutation	
**COLO-704**	*YES*	nc	nc	nc	nc	nc	nc	nc	nc	nc	R130* (Hm)	nc	nc	nc	nc	nc	nc	nc	c.1146delA (het)	Nonsense Mutation	
**COLO-720E**	*NO*	nc	nc	nc	nc	nc	nc	nc	nc	nc	R130* (Hm)	nc	nc	nc	nc	nc	nc	nc	p.A138V (het) / c.1118delA (het)	Nonsense Mutation	
**MCAS**	*YES*	nc	G12D	nc	nc	nc	nc	nc	nc	nc	nc	nc	nc	nc	H1047R	nc	nc	nc	127bp Del, Ex 4	Nonsense Mutation	
**HEY**	*HEY-A1*	nc	G12D	nc	nc	nc	nc	nc	nc	nc	nc	nc	nc	nc	nc	nc	nc	nc	nc	nc	

NC - no change detected
ERBB2 (W746C) - unknown significance not reported in COSMIC
All mutation are heterozygous unless noted as (Hm) = Homozygous/Hemizygous
