# Supplementary material for: Type-Specific Cell Line Models for Type-Specific Ovarian Cancer Research
Source: PLoS One. 2013 Sep 4;8(9):e72162. doi: 10.1371/journal.pone.0072162 (PMC3762837; doi:10.1371/journal.pone.0072162)
Supplement: Table S5 — Mismatch Repair IHC. (PDF) [file pone.0072162.s006.pdf]

Supplemental Table S5: Mismatch Repair IHC

Cell Line	Group 1		Group 2	
	MLH-1	PMS-2	MSH-2	MSH-6
2008	1	1	1	1
A2780	0	0	1	1
CAOV3	1	1	1	1
COLO-704	0	0	1	1
COLO-720E	0	0	1	1
ES-2	1	1	1	1
HEY	1	1	1	1
IGROV1	0	0	0	0
JHOC-5	1	1	1	1
JHOC-7	1	1	1	1
JHOC-9	1	1	1	1
Kuramochi	1	1	1	1
MCAS	1	1	1	1
OV90	N/A	N/A	N/A	N/A
OVCAR-3**	1	1	1	1
OVCAR-4	1	1	1	1
OVCAR-5	1	1	1	1
OVCAR-8	1	1	1	1
OVISE	1	1	1	1
OVMANA	1	1	1	1
OVSAYO	1	1	1	1
OVTOKO	1	1	1	1
RMG-1	1	1	1	1
RMG-2	1	1	1	1
SKOV3**	0	0	1	1
TOV112D	1	1	1	1
TOV21G	0	0	1	1
VOA1056_CL*	1	1	1	1
VOA1072_CL*	1	1	1	1
VOA1312_CL	1	1	1	1
VOA1400_CL*	1	1	1	1
VOA1416_CL*	1	1	1	1

*** results verified in primary tissue AND derivative cell line**

1 - nuclear protein, 0 - no detectable protein (pressumed MMR deficient)

**** reported in Taverna P et al. (Cancer Chemother Pharmacol. 2000;46(6):507-16)**
